# Induction of complex immune responses and strong protection against retrovirus challenge by adenovirus-based immunization depends on the order of vaccine delivery

**DOI:** 10.1186/s12977-017-0336-7

**Published:** 2017-02-06

**Authors:** Meike Kaulfuß, Ina Wensing, Sonja Windmann, Camilla Patrizia Hrycak, Wibke Bayer

**Affiliations:** Institute for Virology, University Hospital Essen, University Duisburg-Essen, Virchowstr. 179, 45147 Essen, Germany

**Keywords:** Adenovirus based immunization, Retrovirus, Vaccine, Human adenovirus, Friend virus, Friend retrovirus, Attenuated retrovirus

## Abstract

**Background:**

In the Friend retrovirus mouse model we developed potent adenovirus-based vaccines that were designed to induce either strong Friend virus GagL_85–93_-specific CD8^+^ T cell or antibody responses, respectively. To optimize the immunization outcome we evaluated vaccination strategies using combinations of these vaccines.

**Results:**

While the vaccines on their own confer strong protection from a subsequent Friend virus challenge, the simple combination of the vaccines for the establishment of an optimized immunization protocol did not result in a further improvement of vaccine effectivity. We demonstrate that the co-immunization with GagL_85–93_/leader-gag encoding vectors together with envelope-encoding vectors abrogates the induction of GagL_85–93_-specific CD8^+^ T cells, and in successive immunization protocols the immunization with the GagL_85–93_/leader-gag encoding vector had to precede the immunization with an envelope encoding vector for the efficient induction of GagL_85–93_-specific CD8^+^ T cells. Importantly, the antibody response to envelope was in fact enhanced when the mice were adenovirus-experienced from a prior immunization, highlighting the expedience of this approach.

**Conclusions:**

To circumvent the immunosuppressive effect of envelope on immune responses to simultaneously or subsequently administered immunogens, we developed a two immunizations-based vaccination protocol that induces strong immune responses and confers robust protection of highly Friend virus-susceptible mice from a lethal Friend virus challenge.

**Electronic supplementary material:**

The online version of this article (doi:10.1186/s12977-017-0336-7) contains supplementary material, which is available to authorized users.

## Background

The development of an effective HIV vaccine has been a priority in retrovirus research since the early days of its discovery more than 30 years ago. Innumerable vector-based vaccine approaches have been developed in pre-clinical models, and many vaccine candidates have been tested in clinical trials, some of them in large phase IIb and III trials. However, in the first efficacy trials hopes were dashed because a protein-based vaccine, although immunogenic, proved completely ineffective [1], and an adenovirus-based vaccine did not result in either protection or reduction of setpoint viral loads and was even associated with an increased HIV infection rate in Ad5 pre-immune, uncircumcised male vaccinees [2, 3]. More encouraging results were obtained in the Thai trial, where a combination of vaccinia-based vectors and the previously phase III-tested protein vaccine conferred modest protection in a low-risk cohort [4]. The development of a potent HIV vaccine is complicated by the lack of a good animal model; the only animal models that support replication of HIV are humanized mice [5] and chimpanzees [6], but the humanized mouse models to date are not very suitable for immunization studies [7]. Therefore, most experimental vaccine studies are carried out in macaques using the related simian immunodeficiency virus (SIV) infection, or as more basic studies in mice, immunizing against HIV proteins without the option of a challenge infection, or working with simple mouse retroviruses. From studies in these animal models, it has been found that only attenuated virus confers truly sterile protection [8–11], but the use of an attenuated HIV for human immunization is regarded by most as too hazardous [12–15]. From experiments performed in the mouse model it could be demonstrated that a complex immune response consisting of CD4^+^ and CD8^+^ T cell and antibody responses is required for complete protection [10, 16].

From studies of human HIV infection and of SIV infection of non-human primates it has been recognized that sterile protection must be achieved to prevent seeding into reservoirs such as latently infected CD4^+^ T cells or immunologically privileged sites, which happens rapidly after infection [17, 18]. It has been demonstrated that an important reservoir are follicular helper CD4^+^ T cells which reside in the immunologically privileged B cell follicle of the lymph nodes where the virus escapes elimination by CD8^+^ T cells [19, 20]. To prevent reservoir seeding, the vaccine-induced immune response, therefore, would have to be very swift; the only vaccine which has been shown to eliminate any detectable virus from reservoirs in the SIV model is a rhesus cytomegalovirus-based vaccine [21, 22] that has been shown to protect via unconventional T cell responses [23, 24].

For the improvement and optimization of adenovirus-based immunization strategies we employ the Friend virus (FV) infection of mice as a retroviral infection model. FV is an immunosuppressive retrovirus complex consisting of the Friend murine leukemia virus (F-MuLV) and the replication-defective, pathogenic Friend spleen focus forming virus [25]. The polycythemic FV complex induces the rapid development of splenomegaly and lethal erythroleukemia in susceptible adult mice, making the FV infection of these mice a very stringent model for vaccine testing, as we have described before [26]. Experiments in the FV model allowed us to develop different strategies to improve the adenovirus-based anti-retroviral immunization. We have reported on the expression-display adenovirus vector design where the vaccine immunogen is not only encoded by the adenoviral vector but also displayed on the adenovirus capsid [27] and the use of select genetic adjuvants such as type I interferons, chemokines and interleukins [28–30] for the induction of improved antibody and CD4^+^ T cell responses. We could also demonstrate the necessity for immunogen modification to allow for the induction of FV-specific CD8^+^ T cells by adenovirus-based immunization [31]. The immunization of highly FV-susceptible mice with these advanced vaccines results in a high degree of protection, with strongly reduced viral loads and protection from FV-induced disease.

Many vaccines have been described before for the immunization against FV, the vaccines tested include attenuated N-tropic F-MuLV-N or FV-N [10, 16, 32, 33], replication-defective [34] or inactivated F-MuLV [35], protein [36, 37] and peptide vaccines [38–40], cell-based vaccines such as FV-derived tumor cells [33] or F-MuLV- or peptide-loaded dendritic cells [41], nano-particle-based vaccines [42], or gene-based vaccines such as DNA-based vectors [43], vaccinia-based vectors [44–46], or the adenovirus-based vectors developed by us [26–31]. While comparisons are sometimes difficult due to the different mouse strains and widely differing FV challenge doses that were employed in the various studies, it has to be noted that until now, only the immunization with attenuated F-MuLV conferred complete protection to FV-susceptible mice.

We describe here our approaches to an optimized combination of our adenovirus-based vaccines for immunization of highly FV-susceptible mice. In order to reach the most extensive immunity, the individual vaccines had to be combined in one immunization regimen to induce all required arms of immunity. Our results show that the order of administration of the individual vaccines is crucial for the vaccination outcome, as the GagL_85–93_-specific CD8^+^ T cell response was greatly diminished if GagL_85–93_/leader-gag encoding vectors were applied together with, or following, env encoding vectors, and the antibody response to an Ad vector delivered immunogen was actually enhanced in adenovirus-experienced mice. We demonstrate that a combination of adenovirus-based vaccines in an optimal vaccination scheme can mediate protection that is comparable to that mediated by a live attenuated Friend murine leukemia virus.

## Methods

### Cells and cell culture

The human embryonic kidney cell line 293 (Microbix Biosystems, Toronto, ON, Canada) was propagated in Dulbecco’s modified Eagle medium (DMEM) with high glucose. A murine fibroblast cell line from *Mus dunni* [47] and the murine hybridoma cell lines 720 [48] and TC31-9C12.C9 [49] (Developmental Studies Hybridoma Bank, IA) were maintained in RPMI medium (Invitrogen/Gibco, Karlsruhe, Germany). Cell culture media were supplemented with 10% heat-inactivated fetal bovine serum (Invitrogen/Gibco) and 50 µg/ml gentamicin. Cell lines were maintained in a humidified 5% CO_2_ atmosphere at 37 °C.

### Adenovirus-based and attenuated retrovirus vaccines

The following vectors have been described before: Ad5.env [26] encodes full-length F-MuLV Env. Ad5.pIXgp70 [27] encodes a fusion protein of the adenovirus capsid protein pIX and F-MuLV Env gp70. Ad5.leader-gag [26] encodes full-length F-MuLV leader-gag protein. Ad5.TxnGagL [31] encodes a fusion protein of the murine cellular protein thioredoxin and the immunodominant F-MuLV CD8^+^ T cell epitope GagL_85–93_. Ad5.GagC1K [31] encodes full-length F-MuLV leader-gag protein with a Y94K mutation. All F-MuLV sequences in the vaccine vectors have been derived from F-MuLV clone FB29 [50]. Ad5.GFP [51] encodes enhanced green fluorescent protein from *Aequorea victoria*. Ad5.ova encodes chicken egg ovalbumin and has originally been published Ad5-ΔGM-OVA [52].

Ad5.empty does not contain any transgenic sequences and was obtained by homologous recombination of pShuttle-CMV with pAdEasy-1 and transfection into 293 cells as described before [53].

All adenoviral vectors were purified with the Vivapure AdenoPACK 100 kit (Vivascience, Hannover, Germany). The adenovirus particle concentrations were determined by spectrophotometry as described previously [54] and calculated as viral particles (vp)/ml. The particle-to-PFU ratio of all vector preparations was ~30:1.

The N-tropic Friend murine leukemia virus (F-MuLV-N) was cultivated on the *Mus dunni* fibroblast cell line and obtained from cell culture supernatant of infected cells.

### Mice

Female CB6F1 hybrid mice (BALB/c x C57BL/6 F1; H-2^b/d^ Fv1^b/b^ Fv2^r/s^ Rfv3^r/s^) and female BALB/c mice were purchased from Charles River Laboratories (Sulzfeld, Germany). All mice were used when they were between 8 and 9 weeks of age.

### Immunization

CB6F1 mice were immunized with 10^9^ vp of the respective adenovirus vaccines subcutaneously into the hind footpads in 50 µl PBS, or intramuscularly in 30 µl PBS. Both administration routes lead to comparable results in our hands (unpublished observation). The amount of virus particles in all groups was maintained equal when some groups received more than one transgene-encoding vector by adding the appropriate amount of empty vector Ad5.empty as needed. When mice were immunized more than once, the immunizations were performed in a three week interval.

Immunization with the attenuated F-MuLV-N was performed by intravenous injection of 10,000 focus forming units in 100 µl PBS.

### FV and challenge infection

Uncloned, lactate dehydrogenase-elevating virus (LDV)-free FV stock was obtained from BALB/c mouse spleen cell homogenate (10%, wt/vol) 14 days post infection with a B cell-tropic, polycythemia-inducing FV complex [55]. CB6F1 mice were challenged by the intravenous injection of 5000 spleen focus-forming units.

### Viremia assay

Ten days post challenge (p.c.), plasma samples from CB6F1 mice were obtained, and viremia was determined in a focal infectivity assay [56]. Serial dilutions of plasma were incubated with *M. dunni* cells for 3 days under standard tissue culture conditions. When cells reached ~100% confluence, they were fixed with ethanol, labeled with F-MuLV Env-specific MAb 720 [48], and then with a horseradish peroxidase (HRP)-conjugated rabbit antimouse Ig antibody (Dako, Hamburg, Germany). The assay was developed using aminoethylcarbazole (Sigma-Aldrich, Deisenhofen, Germany) as substrate to detect foci. Foci were counted, and focus-forming units (FFU)/ml plasma were calculated.

### Infectious center assay

21 days p.c., animals were sacrificed by cervical dislocation, the spleens were removed and weighed, and single-cell suspensions were prepared. Serial dilutions of isolated spleen cells were seeded onto *M. dunni* cells, and cells were incubated under standard tissue culture conditions for 3 days, fixed with ethanol, and stained as described for the viremia assay. Resulting foci were counted, and infectious centers (IC)/spleen were calculated.

### Binding antibody ELISA

For the analysis of F-MuLV-binding antibodies, MaxiSorp ELISA plates (Nunc, Roskilde, Denmark) were coated with whole F-MuLV antigen (5 µg/ml); for the analysis of adenovirus-binding antibodies, plates were coated with 5 µg/ml Ad5.empty. After coating, plates were blocked with 10% fetal calf serum in PBS, and incubated with serum dilutions. Binding antibodies were detected using a polyclonal rabbit-anti-mouse HRP-coupled anti-IgG antibody and the substrate tetramethylbenzidine (TMB+; both Dako Deutschland GmbH, Hamburg, Germany). Sera were considered positive if the optical density at 450 nm was threefold higher than that obtained with sera from naïve mice.

### Complement-dependent F-MuLV-neutralizing antibody assay

To detect F-MuLV-neutralizing antibodies, serial dilutions of heat-inactivated plasma in PBS were mixed with purified F-MuLV and guinea pig complement (Sigma Aldrich, Munich, Germany), incubated at 37 °C for 60 min, and then added to *M. dunni* cells that had been plated at a density of 7.5 × 10^3^ cells per well in 24-well plates the day before. Seventy-two hours later cells were stained as described for the viremia assay. Dilutions that resulted in a reduction of foci by 90% or more were considered neutralizing.

### Tetramer staining of F-MuLV-specific CD4^+^ T cells

F-MuLV-specific CD4^+^ T cells were analyzed in peripheral blood cells two weeks after immunization or 10 days p.c., or in spleen cells three days p.c.; erythrocytes were lysed before the staining when blood samples were used. Cells were stained with an allophycocyanin (APC)-coupled major histocompatibility complex (MHC) class II tetramer (containing the I-Ab-restricted F-MuLV Env_123–141_ epitope EPLTSLTPRCNTAWNRLKL [57]; kindly provided by the MHC Tetramer Core Facility of the National Institutes of Health, National Institute of Allergy and Infectious Diseases, Atlanta, GA), fluorescein isothiocyanate (FITC)–anti-CD11b, peridinin chlorophyll protein (PerCP)–anti-CD43, Brilliant Violet (BV) 510-anti-CD44, BV605-anti-CD4 (Becton–Dickinson, Heidelberg, Germany) and Fixable Viability Dye eFluor 780 (eBioscience, Frankfurt, Germany). Data were acquired on an LSR II flow cytometer (Becton–Dickinson, Mountanview, CA) and analyzed using FlowJo software (Tree Star, Ashton, OR).

### Tetramer staining of F-MuLV-specific CD8^+^ T cells

F-MuLV-specific CD8^+^ T cells were analyzed in peripheral blood two weeks after immunization, or 10 days p.c. After lysis of erythrocytes, blood cells were stained with PE-coupled MHC I tetramer (containing the H-2D^b^ restricted F-MuLV Gag-leader epitope AbuAbulLAbuLTVFL in which cysteine residues of the original amino acid sequence GagL_85–93_ (CCLCLTVFL) were replaced by amino-butyric acid (Abu) to prevent disulfide bonding [58]; MBL, Woburn, MA), PerCP-anti-CD43, eFluor450-anti-CD8, BV510-anti-CD44 (Becton–Dickinson, Heidelberg, Germany) and Fixable Viability Dye eFluor 780 (eBioscience, Frankfurt, Germany). Data were acquired on an LSR II flow cytometer (Becton–Dickinson, Mountanview, CA) and analyzed using FlowJo software (Tree Star, Ashton, OR).

### Flow-cytometric analysis of regulatory T cells

Regulatory T cells were analyzed in popliteal and inguinal lymph nodes draining the site of intramuscular vaccine administration two weeks after the immunization. Lymph node cells were stained with antibodies AlexaFluor680-anti-CD4, FITC-anti-CD44, PE-anti-CD103, PeCy7-anti-CD62L, APC-anti-KLRG1 (Becton–Dickinson, Heidelberg, Germany) and Fixable Viability Dye eFluor 780 (eBioscience, Frankfurt, Germany); after fixation with FoxP3/transcription factor fixation/permeabilization kit (eBioscience, Frankfurt, Germany) cells were stained intracellularly with eFluor450-anti-FoxP3 (Becton–Dickinson, Heidelberg, Germany). Data were acquired on an LSR II flow cytometer (Becton–Dickinson, Mountanview, CA) and analyzed using FlowJo software (Tree Star, Ashton, OR).

### Intracellular cytokine staining

For the analysis of effector molecules of immunogen- or vector-specific CD8^+^ T cells, blood samples were collected and subjected to erythrocyte lysis. Cells were stimulated for 6 h in vitro with 2 µg/ml ova_257–264_ peptide (SIINFEKL), 1 µg/ml Abu-modified GagL_85–93_ peptide (AbuAbuLAbuLTVFL; Abu-modified from the original sequence CCLCLTVFL), or 10 µg/ml Ad5 Hexon_486–494_ (KSYPSNVKI) and DBP_418–426_ (FALSNAEDL) [59] in the presence of 2 µg/ml brefeldin A. Cells were stained with eFluor450-anti-CD8, PerCP-anti-CD43, BV510-anti-CD44, FITC-anti-interferon γ (IFNγ), PE-anti-interleukin 2 and APC-Cy7-anti-tumor necrosis factor α (TNFα; Becton–Dickinson, Heidelberg, Germany).

For the analysis of effector molecules of F-MuLV Env-specific CD4^+^ T cells, blood samples were collected and subjected to erythrocyte lysis; then cells were stimulated in vitro for 72 h with 10 µg/ml of the peptides Env_57–71_ (ETVWAISGNHPLWTW), Env_91–105_ (GLEYRAPYSSPPGPP), Env_415–430_ (KGSYYLVAPAGTMWAC), Env_267–281_ (PRVPIGPNPVLADQL), Env_277–291_ (LADQLSFPLPNPLPK) [60] and Env_123–141_ (EPLTSLTPRCNTAWNRLKL) in the presence of 10 units/ml human interleukin 2 (IL2) (Roche Diagnostics, Mannheim, Germany). Cells were then restimulated with the above peptides in the presence of 10 units/ml human interleukin 2 and 2 µg/ml brefeldin A for an additional 6 h. Cells were stained with BV605-anti-CD4, BV510-anti-CD44, FITC-anti-IFNγ, PE-anti-IL2, PE-Cy7-anti-IL4, APC-anti-IL21, and BV421-anti-IL10.

All data were acquired on an LSR II flow cytometer (Becton–Dickinson, Mountanview, CA) and analyzed using FlowJo software (Tree Star, Ashton, OR).

### Transfer of cells and plasma

For the transfer of CD4^+^ T cells, single cell suspensions were prepared from lymph nodes and spleens, and CD4^+^ T cells were isolated by magnetic bead labelling using a CD4^+^ T cell isolation kit (Miltenyi Biotec, Bergisch-Gladbach, Germany). Successful cell separation was confirmed by flow cytometry, and 10^7^ CD4^+^ cells in 200 µl PBS containing 3 units heparin were injected intravenously into recipient mice.

For the transfer of plasma, blood was collected and immediately mixed with 1.5 units heparin/100 µl, and blood cells were removed by centrifugation at 1000*g* for 10 min. After verification of the anti-adenovirus antibody titer by ELISA, 200 µl plasma were injected intravenously into recipient mice.

To obtain antibody-free plasma, samples were collected as described above, and incubated repeatedly with protein G-Sepharose beads (GE Healthcare Life Sciences, Freiburg, Germany). Removal of any detectable adenovirus-specific antibodies was confirmed by ELISA. Mice received an equal amount, i.e. 200 µl, of the antibody-cleared plasma.

### Statistical analyses

Statistical analyses were performed using the software SigmaStat 3.1 (Systat Software GmbH, Erkrath, Germany), testing with the Mann–Whitney Rank Sum test or Student’s t-test for the comparison of two groups or the Kruskal–Wallis one-way analysis of variance on ranks and Student–Newman–Keuls (equally sized groups) or Dunn’s (unequally sized groups) multiple comparison procedure for the comparison of three or more groups. Analysis of statistical power was performed using the software GPower3.1 [61].

## Results

### Combination of Env and Gag encoding vectors results in loss of GagL_85–93_-specific CD8^+^ T cell responses

We have described before that vaccination with the vaccines Ad.TxnGagL or Ad.Gag_C1K_ induce strong CD8^+^ T cell responses to the immunodominant F-MuLV CD8^+^ T cell epitope GagL_85–93_ and induces a high degree of protection in highly FV-susceptible mice [31]. On the other hand, we have developed vectors and vaccine combinations that confer similarly strong protection by the induction of F-MuLV-specific antibodies and CD4^+^ T cells [27–30]. While the immune protection conferred by these different vaccines is very good and protects highly susceptible mice from FV-induced disease, protection is not complete and most mice still harbor virus, albeit at low levels.

**Fig. 1:**
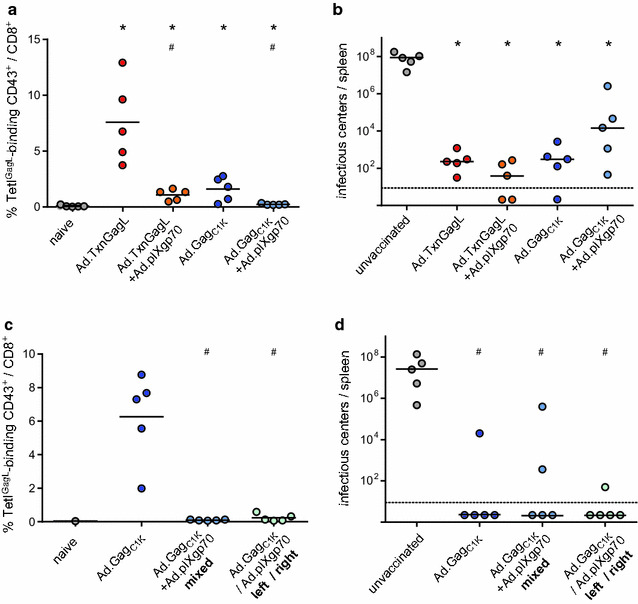
Induction of GagL_85–93_-specific CD8^+^ T cell responses by combined Ad-based vaccines. **a**, **b** CB6F1 mice were immunized twice in a 3-week interval by subcutaneous injection with 10^9^ vp of the indicated vaccines, the groups that received only one transgene-encoding vector received an equal amount of Ad.empty. Vaccines were mixed and injected into both hind footpads. **c**, **d** CB6F1 mice were immunized twice in a three-week interval by subcutaneous injection of 10^9^ vp of the indicated vaccines, the two vaccines Ad.Gag_C1K_ and Ad.pIXgp70 were either mixed and injected into both hind footpads (“mixed”), or the two vaccines were kept separate and injected into one footpad each (“left/right”). GagL_85–93_-specific CD8^+^ T cell responses were analyzed in peripheral blood samples two weeks after the second immunization (**a**, **c**). Three weeks after the second immunization, mice were infected with FV, and the viral load in spleens was analyzed three weeks after the FV challenge (**b**, **d**). The data shown were obtained in two independent experiments. *Each dot* represents one mouse, *lines* indicate mean (**a**, **c**) or median (**b**, **d**) values, *dashed lines* indicate the limit of detection. Data were analyzed by Kruskall–Wallis One Way Analysis of Variance on Ranks and Student–Newman–Keuls multiple comparison procedure; significant differences (*P* < 0.05) compared to unvaccinated mice are indicated by *, significant differences compared to mice vaccinated with the respective single vaccine are indicated by #. Sufficient statistical power was verified [SP = 0.89 (**a**), 1.0 (**b**), 0.89 (**c**), 0.99 (**d**)]

To further increase the effectivity of our adenovirus-based vaccination regimen, we combined the two CD8^+^ T cell inducing vaccines Ad.TxnGagL and Ad.Gag_C1K_ with the expression-display vector Ad.pIXgp70, which encodes and displays a fusion protein of the adenovirus capsid protein pIX and the F-MuLV envelope gp70 and induces strong antibody and CD4^+^ T cell responses. CB6F1 mice were immunized twice with Ad.TxnGagL or Ad.Gag_C1K_ alone or in combination with Ad.pIXgp70, and two weeks after the second immunization the GagL_85–93_-specific CD8^+^ T cell response was analyzed by MHC I tetramer staining. Mice that were immunized with Ad.TxnGagL or Ad.Gag_C1K_ alone mounted a GagL_85–93_-specific CD8^+^ T cell response that, while higher in the Ad.TxnGagL group as seen and reported before [31], was easily detectable in both groups; co-immunization with Ad.pIXgp70 however led to a significant reduction of the GagL_85–93_-specific CD8^+^ T cell response (Fig. 1a; *P* < 0.05). Accordingly, when the viral loads in spleens of mice three weeks after an FV challenge infection were analyzed, we found no improved protection in the mice that were immunized with the vaccine combinations (Fig. 1b).

The dampening effect of the F-MuLV envelope on the response to other immunogens is not exclusively observed with other retroviral proteins but could also be demonstrated when mice were immunized with an ovalbumin encoding adenoviral vector, where we found the CD8^+^ T cell response to the ovalbumin epitope ova_257–264_ significantly reduced by co-adminstration of Ad.Env (Additional file 1; *P* < 0.05).

This reduction of CD8^+^ T cell responses by the co-administration of Ad.Env suggested that immunodominance effects might be at play, therefore we analyzed if the abrogation of the CD8^+^ T cell induction was dependent on colocalization of the two vaccines. We immunized mice with Ad.Gag_C1K_ and Ad.pIXgp70 and applied the two vaccines either as a mixture, or separately, one into each hind leg. The CD8^+^ T cell response to GagL_85–93_ was again analyzed by MHC I tetramer staining two weeks after immunization, and it was apparent that both groups immunized with the two vectors had failed to mount an appropriate GagL_85–93_-specific CD8^+^ T cell response, in stark contrast to the control group that received the Ad.Gag_C1K_ vaccine alone (Fig. 1c; *P* < 0.05). Interestingly, the protection conferred to all three groups was equally strong, and many animals had undetectable viral loads (Fig. 1d).

### The order of vaccine administrations is crucial for the induction of CD8^+^ T cells

**Fig. 2:**
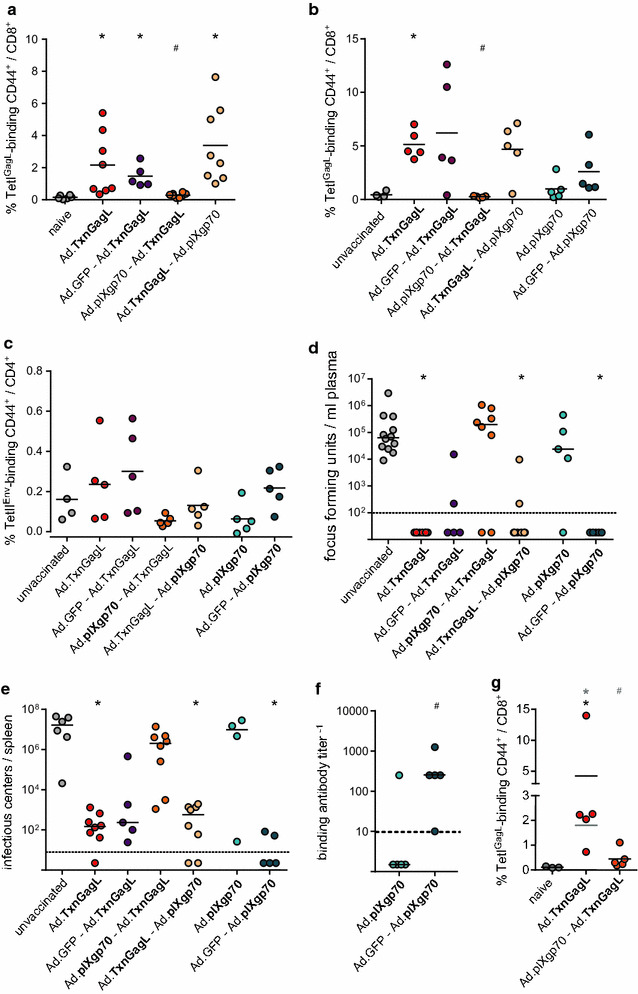
Immune responses and protection induced by sequential Ad-based vaccination. CB6F1 mice were immunized at two time points with 10^9^ vp of the indicated vaccines; immunizations were performed in a three-week interval, only one vaccine was used in each immunization. The GagL_85–93_-specific CD8^+^ T cell response was analyzed by MHC I tetramer staining of peripheral blood cells two weeks after the second immunization (**a**). Three weeks after completion of the immunizations, mice were challenged with FV, and 10 days after FV infection GagL_85–93_-specific CD8^+^ T cell responses (**b**) and Env_123–141_-specific CD4^+^ T cells (**c**) were analyzed in peripheral blood cells by MHC I and MHC II tetramer staining, respectively. FV load in plasma was analyzed 10 days (**d**), viral load in spleens three weeks after FV infection (**e**). F-MuLV-binding antibody titers were analyzed in sera collected two weeks after completion of the immunizations (**f**). In another experiment, CB6F1 mice were immunized once with Ad.TxnGagL, with or without an immunization with Ad.pIXgp70 three months earlier. GagL_85–93_-specific CD8^+^ T cell responses were analyzed in peripheral blood by MHC I tetramer staining two weeks after Ad.TxnGagL immunization (**g**). The data shown were obtained in two independent experiments (**a**, **d**, **e**) or one experiment (**b**, **c**, **f**, **g**). *Each dot* represents one mouse, *lines* indicate mean [**a**–**c**, **g** (*grey line*: mean value calculated with exclusion of outlier)] or median (**d**–**f**) values, *dashed lines* indicate detection limits. Data were analyzed by Kruskall–Wallis One Way Analysis of Variance on Ranks and Dunn’s multiple comparison procedure (**a**–**e**, **g** without exclusion of outlier from the calculation), One Way Analysis of Variance and Student–Newman–Keuls multiple comparison procedure (**g** with exclusion of outlier from the calculation) or Mann–Whitney Rank Sum Test (**f**). Significant differences (*P* < 0.05) compared to unvaccinated mice are indicated by *, significant differences compared to mice vaccinated with the respective single vaccine are indicated by # (**a**, **b**: compared to Ad.TxnGagL; F: compared to Ad.pIXgp70); *black symbols* indicate significant differences calculated including all values, *grey symbols* indicate significant differences after exclusion of the outlier (**g**). Sufficient statistical power was verified [SP = 0.99 (**a**), 0.99 (**b**), 0.81 (**c**), 0.99 (**d**), 0.99 (**e**), 1.0 (**f**), 0.99 (**g**)]

As the spatial separation of the two vaccines did not result in the induction of CD8^+^ T cells, we immunized mice once with Ad.TxnGagL and Ad.pIXgp70, one vaccine at a time, using either Ad.TxnGagL or Ad.pIXgp70 first, followed three weeks later by the other vaccine (see Additional file 2 for the vaccination scheme). When the GagL_85–93_-specific CD8^+^ T cell response was analyzed two weeks after the second immunization, we found an expectedly high frequency of GagL_85–93_-specific CD8^+^ T cells in mice that had received only one immunization with Ad.TxnGagL, and also the control group that had received a GFP-encoding vector as the first immunization showed an equally high GagL_85–93_-specific CD8^+^ T cell response (Fig. 2a). However, mice that had received Ad.pIXgp70 as the first vaccine followed by Ad.TxnGagL mounted a gravely reduced GagL_85–93_-specific CD8^+^ T cell response (*P* < 0.05). Mice that had received Ad.TxnGagL first, followed by Ad.pIXgp70, showed a GagL_85–93_-specific CD8^+^ T cell response that was comparable to that seen in mice immunized with Ad.TxnGagL alone, demonstrating that the subsequent immunization with Ad.pIXgp70 did not abrogate the CD8^+^ T cell response induced by the first immunization. Interestingly, this also demonstrates that the GagL_85–93_-specific CD8^+^ T cell population had not contracted noticeably at this time point five weeks after the immunization.

To analyze the GagL_85–93_-specific CD8^+^ T cell response in more detail, we restimulated cells from immunized mice with GagL_85–93_ peptide and subjected them to intracellular cytokine staining (Additional file 3). Stimulation of cells from unvaccinated mice resulted in a low background staining for IFNγ, TNFα and IL2; cells from mice immunized first with Ad.pIXgp70 followed by Ad.TxnGagL gave a similar picture yet with a very small population of cells producing two cytokines. On the other hand, if mice received Ad.TxnGagL either as a single immunization or as the first vaccine followed by Ad.pIXgp70, the frequency of cytokine-producing cells was strongly increased with almost half of the cells producing more than one cytokine.

Three weeks after the completed immunization, mice were challenged with Friend virus, and ten days later blood samples were collected for the analysis of viral loads and of CD4^+^ and CD8^+^ T cell responses. The MHC I tetramer staining showed the same trends as observed before the challenge infection, with a still severely impaired GagL_85–93_-specific CD8^+^ T cell response in the mice immunized with Ad.pIXg70 followed by Ad.TxnGagL, which was comparable to that observed in unvaccinated mice, demonstrating that there was no rapid anamnestic proliferation of vaccine-induced GagL_85–93_-specific CD8^+^ T cells (Fig. 2b). The MHC II tetramer staining showed no significant differences between the vaccinated groups at this time point (Fig. 2c). The mice that mounted strong GagL_85–93_-specific CD8^+^ T cell responses showed a strong reduction in viral loads in the plasma on day 10 and in the spleens on day 21 after FV challenge infection (Fig. 2d, e); mice that were immunized with Ad.pIXgp70 followed by Ad.TxnGagL had mostly very high viral loads. The mice that had received a single immunization with Ad.pIXgp70 had similarly high viral loads that were not significantly lower than in unvaccinated mice, however, if mice had received an injection of the control vector Ad.GFP before the immunization with Ad.pIXgp70, they surprisingly showed a very strong protection from FV infection, with undetectable viremia and very low, if detectable, spleen viral load. As we had not seen significant differences in the cellular immune responses of mice immunized with Ad.pIXgp70 with or without a prior immunization with Ad.GFP (Fig. 2b, c), we analyzed F-MuLV-binding antibodies in serum samples collected two weeks after the Ad.pIXgp70 immunization (Fig. 2f). While all but one mice that had received a single injection of Ad.pIXgp70 without prior immunization failed to mount detectable antibodies at this time point, mice that had been injected with Ad.GFP before receiving Ad.pIXgp70 had a significantly higher binding antibody response (*P* < 0.05).

To further characterize the suppressive effect of Ad.pIXgp70 on the subsequent Ad.TxnGagL immunization, we investigated if the effect would persist over a longer time period. Therefore, mice were immunized once with Ad.pIXgp70, and immunized 15 weeks later with Ad.TxnGagL. When we analyzed the GagL_85–93_-specific CD8^+^ T cell response two weeks later, control mice that had been naïve prior to Ad.TxnGagL immunization showed a significant induction of specific CD8^+^ T cells, while the mice that had been pre-immunized with Ad.pIXgp70 were still mostly unable to mount a GagL_85–93_-specific CD8^+^ T cell response (Fig. 2g), clearly demonstrating that immunization with an F-MuLV Env encoding vector leads to long-term modification of the response to a subsequent immunization.

### Immunization with envelope encoding adenovirus vectors does not lead to expansion of regulatory T cells

**Fig. 3:**
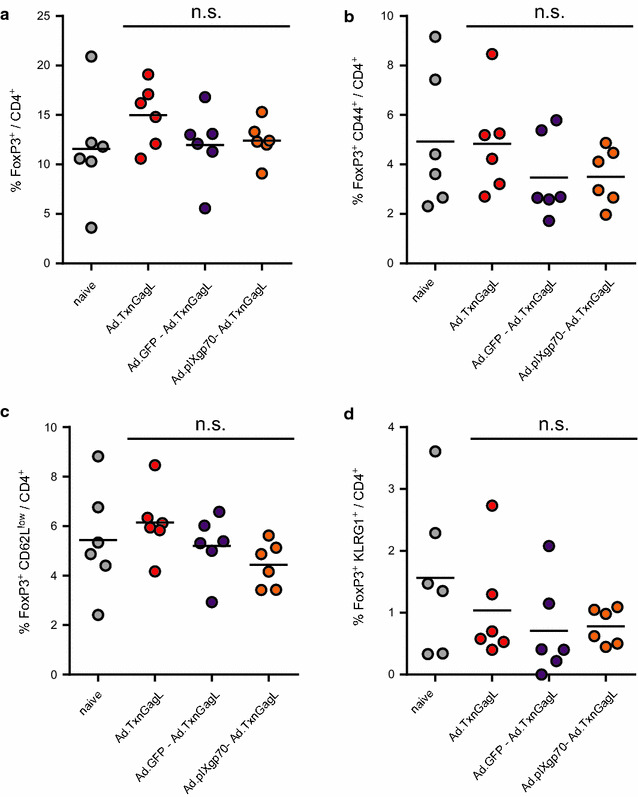
Induction of regulatory T cells by Ad-based vaccination. CB6F1 mice were immunized with 10^9^ vp Ad.TxnGagL, either as a single immunization or after pre-immunization with 10^9^ vp Ad.GFP or Ad.pIXgp70. Three days after the Ad.TxnGagL immunization, the frequencies of regulatory FoxP3^+^ CD4^+^ T cells (**a**), of CD44^+^ FoxP3^+^ CD4^+^ T cells (**b**), of CD62L^low^ FoxP3^+^ CD4^+^ T cells (**c**), and of KLRG1^+^ FoxP3^+^ CD4^+^ T cells (**d**) were analyzed in draining lymph nodes. The data shown were obtained in two independent experiments. *Each dot* represents one mouse, *lines* indicate mean values. Data were analyzed by Kruskall–Wallis One Way Analysis of Variance on Ranks and Dunn’s multiple comparison procedure, *n.s.* not significant (*P* > 0.05)

As the impact of F-MuLV Env encoding vectors on subsequent immunizations was strong and long-lasting, we suspected a potential role for regulatory T cells. Therefore, we repeated the sequential immunization with Ad.pIXgp70 and Ad.TxnGagL and analyzed the frequency of regulatory FoxP3^+^ CD4^+^ T cells, as well as their activation status, in draining lymph nodes three days after the Ad.TxnGagL immunization. When we compared the frequency of regulatory T cells in the CD4^+^ T cell subset (Fig. 3a), there was no significant increase in the mice that had been immunized with Ad.pIXgp70 before the immunization with Ad.TxnGagL compared to naïve mice, pre-naïve Ad.TxnGagL immunized mice or mice that had been pre-immunized with Ad.GFP. Also the activation status of the regulatory CD4^+^ T cells was not significantly altered by any of the immunizations as analyzed by expression of CD44, CD62L, or KLRG1 (Fig. 3b–d). Thus, we did not find any evidence for a contribution of regulatory CD4^+^ T cells to the immunosuppression observed after F-MuLV Env immunization.

### Immunization with envelope encoding adenovirus vectors leads to higher antibody titers in adenovirus-experienced mice

**Fig. 4:**
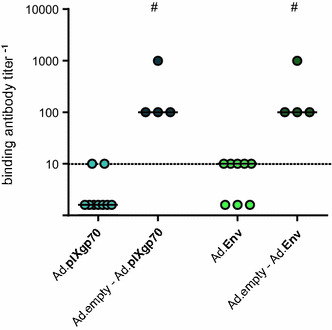
Env-specific binding antibodies after Ad-based vaccination of Ad-experienced mice. CB6F1 mice were immunized with 10^9^ vp of the indicated envelope vaccines, with or without immunization with 10^10^ vp Ad.empty three weeks prior as indicated. F-MuLV-binding antibody titers were analyzed two weeks after completion of the immunization. The data shown were obtained in two independent experiments, using 2–4 mice per group per experiment. *Each dot* represents one mouse, *lines indicate* median values, *dashed lines* indicate the limit of detection. Data were analyzed by Mann–Whitney Rank Sum Test. Significant differences (*P* < 0.05) compared to mice vaccinated only with the envelope vaccine are indicated by #. Sufficient statistical power was verified (SP = 0.99)

We followed up on the surprising finding that the mice that had received Ad.GFP before the actual immunization with Ad.pIXgp70 mounted higher antibody responses, enquiring if the effect would persist if mice received a higher dose of control Ad vector before immunization with Ad.pIXgp70 or with the conventional envelope-encoding Ad.Env, using 10^10^ vp of Ad.empty for the pre-treatment. Two weeks after the immunization with Ad.pIXgp70 or Ad.Env we analyzed the F-MuLV-binding antibody response (see Additional file 4A for the experimental layout); while mice that had received only the envelope-encoding vectors had not mounted a significant antibody response at the time, those mice that had been injected with Ad.empty before the actual immunization with Ad.pIXgp70 or Ad.Env had significantly higher antibody titers (Fig. 4). To investigate if this effect is limited to envelope protein, we performed a similar experiment with a leader-gag encoding Ad vector; again, mice that had been pre-treated with an empty Ad vector mounted significantly higher binding antibodies than pre-naïve mice (Additional file 5).

**Fig. 5:**
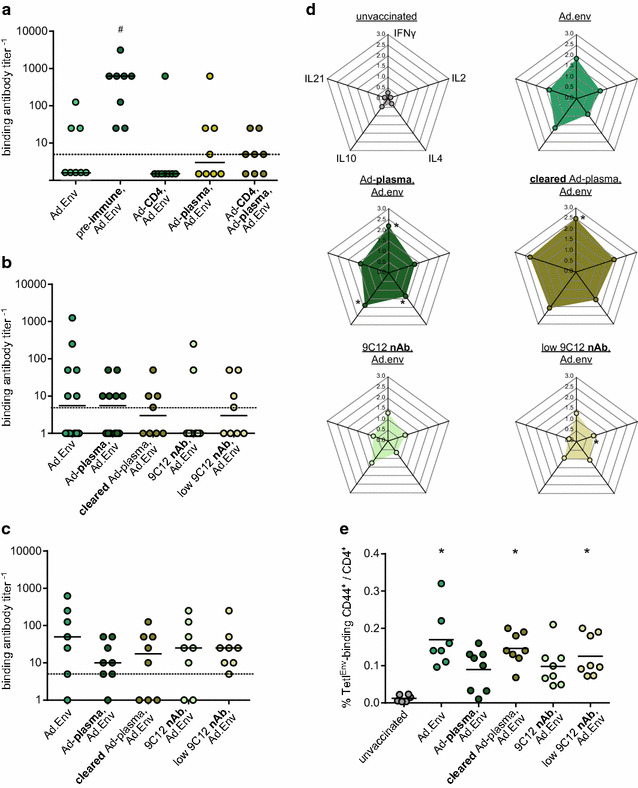
Influence of pre-existing immunity on Ad-based induction of Env-specific binding antibodies and CD4^+^ T cells. CB6F1 mice were immunized with Ad.Env without pre-treatment, three weeks after two pre-immunizations with 10^9^ vp Ad.empty in three-week intervals, or one day after transfer of 10^7^ CD4^+^ T cells, or 200 µl plasma, or both from Ad pre-immune mice. Binding antibodies were analyzed two weeks after immunization with Ad.Env (**a**). In another transfer experiment, mice received plasma or antibody-cleared plasma from Ad pre-immune mice, or the Ad-neutralizing antibody 9C12 before immunization with 10^9^ vp Ad.Env. Binding antibodies were analyzed two weeks after one (**b**) or two immunizations with Ad.Env (**c**). Two weeks after the first Ad.Env immunization, blood cells were stimulated with Env-derived peptides and cytokine production by CD4^+^ T cells was analyzed by flow cytometry (**d**). Three weeks after the second Ad.Env immunization, mice were infected with FV and three days later, Env_123–141_-specific CD4^+^ T cells were analyzed by MHC II tetramer staining (**e**). The data shown were obtained in two or three (**b**: Ad.Env and Ad-plasma,Ad.Env) independent experiments with four mice per group. *Each dot* represents one mouse (**a**–**c**, **e**) or mean values (**d**), *lines* indicate median (**a**–**c**) or mean (**e**) values, *dashed lines* indicate the limits of detection. Axes of the radar charts all have the scale as indicated for the first axis; the order of data shown in all plots is as indicated for the first radar chart (**d**). Data were analyzed by Kruskall–Wallis One Way Analysis of Variance on Ranks and Dunn’s multiple comparison procedure. Significant differences (*P* < 0.05) compared to unvaccinated mice are indicated by *, significant differences compared to mice vaccinated with Ad.Env are indicated by #. Sufficient statistical power was verified [SP = 0.99 (**a**), 1.0 (**b**), 1.0 (**c**), 0.93 (**d**: IFNγ), 0.99 (**e**)]

It is widely acknowledged that in pre-immune individuals, the presence of anti-adenovirus neutralizing antibodies abrogates the induction of transgene-specific immune responses; the induction of anti-vector immunity by repeated immunizations also diminishes the induction of transgene-specific immunity in vector-based immunization (reviewed in [62]). We established a pre-immunization protocol using two immunizations with 10^9^ vp of the control vector Ad.empty that leads to induction of Ad-specific CD8^+^ T cell responses and Ad-specific antibodies (Additional file 6A, B), and results in complete loss of induction of transgene-specific CD8^+^ T cell responses to a subsequent immunization with Ad.TxnGagL (Additional file 6C). Interestingly, when we pre-immunized mice in this way and subsequently immunized them with Ad.Env, their antibody response was significantly higher than after an immunization without prior adenovirus injection (Fig. 5a). We also performed cell and plasma transfer from pre-immunized mice to naïve mice before Ad.Env immunization (see Additional file 4B for the experimental layout), but could not demonstrate an enhancing effect by either transfer; but as all but one mice that had received CD4^+^ T cells from Ad.empty pre-immunized mice had no detectable anti-F-MuLV antibody titer, the involvement of CD4^+^ T cells seems unlikely. Importantly, the transfer of Ad-immune plasma did not lead to an abrogation of Env-specific antibody induction.

To dissect the role of different plasma components, we performed another transfer experiment, where we addressed the role of neutralizing antibodies and cytokines separately. For this, plasma from Ad pre-immunized mice with high titers of binding antibodies was cleared of antibodies by repeated incubation with protein G-Sepharose; on the other hand, we used the neutralizing hexon-specific antibody 9C12 [63] either at a concentration resulting in a titer similar to the whole anti-Ad antibody titer found in pre-immunized mice, or at a 100-fold reduced concentration, which we found to represent the titer of neutralizing antibodies in plasma samples from pre-immunized mice (data not shown). We analyzed the binding antibody titers two weeks after one (Fig. 5b) or two immunizations (Fig. 5c) with Ad.Env, and found no significant differences between the groups, importantly including the mice that were treated with the neutralizing 9C12 antibody, indicating that priming of the antibody response was not impaired by the presence of Ad-specific antibodies, and expansion upon boost was equally efficient in all groups. When we analyzed the cytokine production by Env-specific CD4^+^ T cells after the first Ad.Env immunization, we found that the mice immunized with Ad.Env produced IFNγ, slightly less IL10 and low levels of interleukins 2, 4 and 21 (Fig. 5d). The induction of cytokine-producing cells was not significantly changed by transfer of Ad-immune plasma prior to Ad.Env immunization, and slightly enhanced when antibody-cleared plasma was transferred. Transfer of the neutralizing antibody 9C12 resulted in a slight trend to lower cytokine production levels, which was also observed when a low dose of 9C12 was transferred. We also analyzed the frequency of Env-specific CD4^+^ T cells by MHC II tetramer staining three days after FV challenge infection, and found a significant induction of Env-specific CD4^+^ T cells in mice immunized with Ad.Env without pre-treatment as well as in mice that had received antibody-cleared plasma or a low dose of the neutralizing antibody 9C12 before Ad.Env immunization, and the frequency was only slightly reduced in mice that had received adenovirus-immune plasma or a high dose of the adenovirus-neutralizing antibody (Fig. 5e).

Overall, our results demonstrate that neither the induction of antibodies nor of CD4^+^ T cells is severely impacted by transfer of either pre-immune serum, the cytokines contained therein, or Ad-neutralizing antibodies.

### Combining adenovirus-based vaccines to induce strong protection against high-dose Friend virus infection

We learned from our experiments that we have to immunize mice with the CD8^+^ T cell inducing vaccine first, before immunizing with Env encoding vectors, in order to elicit the full width of immune responses that the individual vaccines are capable of inducing. Therefore we developed a sequential vaccination protocol that encompasses one immunization with Ad5.TxnGagL and Ad5.Gag_C1K_, reasoning that the combination of the two vaccines would induce strong GagL_85–93_-specific CD8^+^ T cell as well as Gag-specific CD4^+^ T cell responses, followed by one immunization with Ad5.pIXgp70, which should result in appreciable antibody levels after a single immunization in the Ad5-experienced mice. We performed this experiment side-by-side with an immunization with attenuated helper virus F-MuLV-N. This N-tropic F-MuLV is highly attenuated in the CB6F1 mice (Fv1^b/b^) employed in our immunization studies and has been described to confer complete protection from a subsequent challenge infection even in highly susceptible mice [10, 16, 32] and can therefore be considered as the gold standard that a vector-based immunization has to meet.

**Fig. 6:**
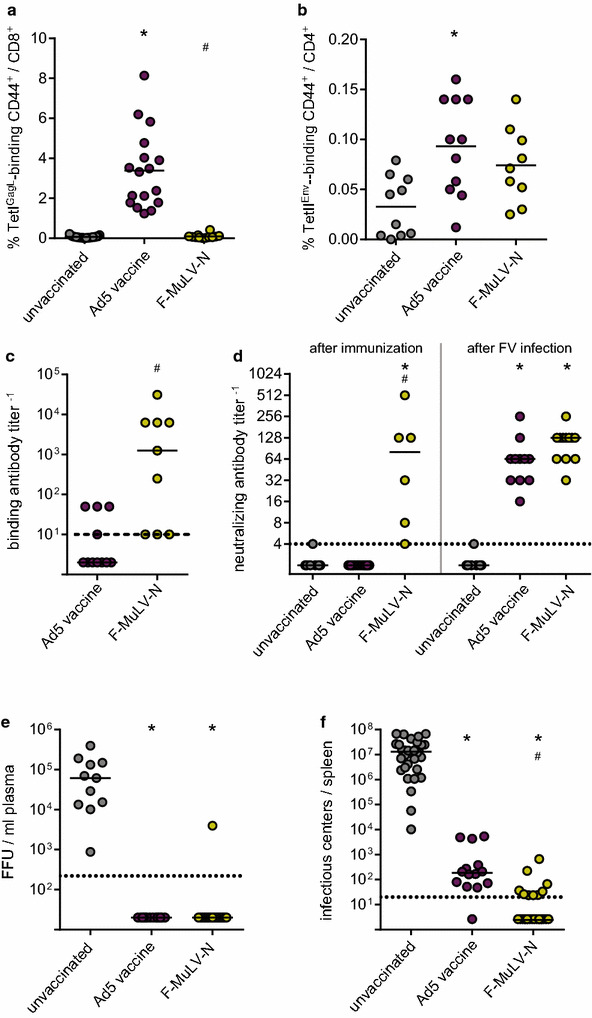
Comparison of immune responses and protection induced by a combined Ad-based vaccine and the attenuated retrovirus F-MuLV-N. CB6F1 mice were either immunized with an Ad based vaccine, consisting of an immunization with 10^9^ vp each of Ad.TxnGagL and Ad.Gag_C1K_, followed by an immunization three weeks later with 10^9^ vp Ad.pIXgp70, or mice were immunized with 10 000 FFU of the N-tropic F-MuLV-N. 6 weeks after the first immunization, mice were challenged with 5000 SFFU FV complex. The frequencies of GagL_85–93_-specific CD8^+^ T cells (**a**) and Env_123–141_-specific CD4^+^ T cells (**b**) were analyzed by MHC I and MHC II tetramer staining, respectively, five weeks after initialization of the immunizations (i.e. 2 weeks after Ad.pIXgp70 immunization); the binding antibody response was analyzed at the same time point (**c**). Neutralizing antibodies were quantified both five weeks after initialization of the immunizations (**d**, left) and 10 days after the FV challenge infection (**d**, right). The viral load in plasma was analyzed 10 days after FV challenge infection (**e**), the viral load in spleens was analyzed three weeks after the FV challenge infection (**f**). The data shown were acquired in three (**b**–**d**) to five independent experiments (**a**, **e**, **f**) using three to five mice per group per experiment. *Each dot* represents one mouse, *lines* indicate mean (**a**, **b**) or median (**c**–**f**) values, *dashed lines* indicate the limits of detection. Data were analyzed by Kruskall–Wallis One Way Analysis of Variance on Ranks and Dunn’s multiple comparison procedure (**a**, **b**, **d**–**f**) or by Mann–Whitney Rank Sum Test (**c**). Significant differences (*P* < 0.05) compared to unvaccinated mice are indicated by *, significant differences compared to mice vaccinated with the Ad-based vaccine are indicated by #. Sufficient statistical power was verified [SP = 0.99 (**a**), 0.88 (**b**), 1.0 (**c**), 0.99 (**d** left), 1.0 (**d** right), 0.99 (**e**), 0.99 (**f**)]

When we analyzed the immune response to our vaccination regimens one week before the FV challenge infection, we confirmed the strong induction of GagL_85–93_-specific CD8^+^ T cells by our Ad-based vaccine (Fig. 6a), and a slight induction of Env_123–144_-specific CD4^+^ T cells (Fig. 6b); interestingly, there were no significant levels of either GagL_85–93_-specific CD8^+^ or Env_123–144_-specific CD4^+^ T cell responses in F-MuLV-N immunized mice. When we analyzed the F-MuLV-binding antibody titers on the other hand, mice that had been immunized with F-MuLV-N had significantly higher binding antibody levels than Ad immunized mice (Fig. 6c), and exhibited substantial neutralizing antibody levels before challenge infection (Fig. 6d left panel). After the FV challenge infection, also the Ad5 immunized mice mounted a robust neutralizing antibody response, indicating efficient priming by the vaccine (Fig. 6d, right panel). Even though the immune responses in the two immunization groups differed remarkably, all immunized mice were able to tightly control a high dose FV challenge infection, with undetectable viral loads in plasma on day 10 in all but one F-MuLV-N-immunized mice (Fig. 6e) and very low or undetectable viral loads in spleens on day 21 after FV challenge (Fig. 6f).

## Discussion

Our data clearly demonstrate the potency of an adenovirus-based immunization that confers strong protection from FV infection in highly susceptible mice, and highlight the important role of immunogen interference effects. The finding that the F-MuLV envelope exerts a potent suppression on the immune response to other immunogens is very intriguing, and the underlying mechanism is still unclear. Retroviral envelope proteins are known to contain an immunosuppressive domain in the transmembrane envelope subunit that is conserved across a wide range of exogenous and endogenous retroviruses (reviewed in [64]), and it has been suggested that altered cytokine expression profiles, including upregulated levels of IL6 and IL10 but also decreased levels of IL2, underlie the suppressive effect [65]. In our experiments, we could demonstrate IL10 production also by Env-specific CD4^+^ T cells. However, the immunosuppressive domain is located in the transmembrane envelope protein, whereas the suppressive activity observed in our experiments was also exerted by gp70, i.e. the surface envelope protein, alone. There have been few publications until now that report a similar effect in DNA immunizations in mice or macaques; it was demonstrated that HIV Env gp120, but not SIV Env gp130 or EIAV Env gp90, abrogated CD8^+^ T cell responses to HIV Gag gp55 [66], others found similar effects for HIV Env and Gag and showed that a reduction of the Env plasmid [67], spatial separation of the Env and Gag plasmids or mutation of an Env epitope [68] led to the rescue of Gag-specific CD8^+^ T cell induction, suggesting epitope competition as an underlying mechanism. Others found a reduction of HIV-specific CD8^+^ T cell responses only when the vectored vaccine was combined with an adjuvanted env gp120 protein vaccine, but not when the vaccine was combined with a vectored gp120 vaccine, but it is unclear if this effect was mediated by env gp120 or the adjuvant [69]. It seems unlikely that epitope competition is the cause for the immunosuppression described here for F-MuLV Env; while we cannot exclude the presence of any CD8^+^ T cell epitopes in F-MuLV Env, the only known CD8^+^ T cell epitope of FV is the H-2D^b^ restricted GagL_85–93_ epitope [58], and while new CD4^+^ T cell epitopes were described recently [60], numerous attempts by us and other groups, using spleen cells from FV-infected mice or from mice immunized with Ad- or plasmid DNA-based vectors, have failed to identify any further epitopes (unpublished data). Ad5 capsid proteins are very immunogenic and can have an adjuvant effect on encoded transgenes or co-delivered substances [70–73], but the Ad5 proteins also contain CD8^+^ T cell epitopes that compete with transgene-derived epitopes [74, 75], therefore the emergence of subdominant epitopes from proteins when expressed from Ad5 vectors seems rather unlikely. Furthermore, spatial separation of the Env and Gag vaccines did not rescue GagL_85–93_-specific CD8^+^ T cell induction, and the suppressive effect was maintained when the Env immunization preceded the Gag immunization, even by as much as three months. While the lasting suppression of CD8^+^ T cell responses to a subsequent immunization suggests an adaptive process, we did not find a significant expansion or activation of regulatory T cells or an altered induction of adenovirus-specific antibodies (data not shown) upon immunization with an envelope-encoding vector compared to other adenovirus vectors. The immediacy of the suppressive effect, which is not relieved by spatial separation, suggests a systemic mechanism like altered cytokine levels; as its lasting nature argues for an adaptive process, our findings may imply a role of cytokine producing cells of the adaptive immune system. It has been shown that the induction of immune responses by adenovirus-based vaccines relies on CD4^+^ T cell help [76–78]; furthermore, HIV gp120 immunization was demonstrated to introduce a Th2 bias via the stimulation of IL10 [79]; thus, a skewed helper T cell profile might underlie the suppression of CD8^+^ T cell induction by a subsequent adenovirus-based immunization. Our findings could have wider implications if they would apply to retroviral envelope proteins in general, and therefore warrant further investigation, both with regard to the mechanism and to their transferability.

To our knowledge, this is the first report on enhanced antibody responses to adenovirus-delivered immunogens in adenovirus-experienced recipients. It is widely accepted that pre-existing anti-adenovirus immunity leads to a loss of efficacy of adenovirus-based vaccines (reviewed in [80]). In many studies however, the focus of the analyses was on cellular immune responses; when antibody responses were analyzed, some studies showed no influence of pre-immunization on the induction of transgene-specific antibody responses by adenovirus-based immunization [81], while other studies reported a reduction or even complete abrogation of transgene-specific immune responses, including antibody responses [82, 83]. Differences in the effects observed here and in other studies may be due to different levels of anti-adenovirus immunity; however, also in our experiments we worked with a pre-immunization schedule that would abrogate induction of transgene-specific CD8^+^ T cells by a subsequent adenovirus-based immunization, indicating that mice had mounted a relevant level of adenovirus-specific immunity. It has been reported for bacterial vector based immunization that a pre-existing immunity against the vector can lead to improved transgene-specific antibody responses (reviewed in [84]). In a study of a Salmonella enterica-based vaccine, the authors found improved immune responses in pre-immune mice, and attributed the enhanced efficacy to an antibody-facilitated uptake by macrophages [85]. While our transfer experiments did not clarify the mechanism underlying the enhanced antibody levels in adenovirus-experienced mice, they did show that adenovirus-specific antibodies in particular did not have an abrogating effect on the adenovirus-based immunization, as might be expected. Analyzing the contribution of isolated components of the immune response may not be a feasible approach to fully grasp the complexity of the immune response in an adenovirus-experienced recipient, and further research is needed to understand the immune enhancement in adenovirus-experienced mice that we describe here.

**Fig. 7:**
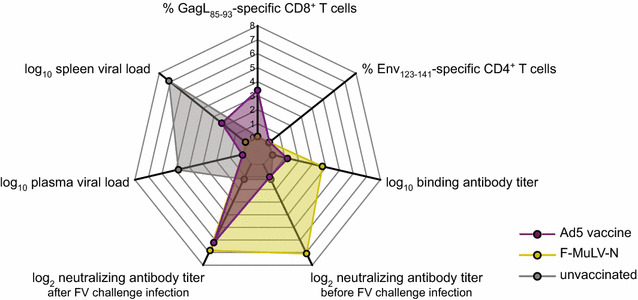
Summary of the immunization experiments with the combined Ad-based vaccine and F-MuLV-N. The radar chart represents the results from Fig. 6, showing (clockwise from the top) the mean percentage of GagL_85–93_-specific CD8^+^ T cells (MHC I Tet^+^ CD44^+^/CD8^+^), the mean percentage of Env_123–141_-specific CD4^+^ T cells (MHC II Tet^+^ CD44^+^/CD4^+^), the mean log_10_ binding antibody titer, the mean log_2_ neutralizing antibody titer before, and after, FV challenge infection, and the median viral loads in plasma on day 10, and in spleens on day 21 after FV challenge infection

When we combined our adenovirus based Env and Gag vaccines, we could show that in spite of the lack of GagL_85–93_-specific CD8^+^ T cell responses, mice were fairly strongly protected from the high dose FV challenge, which is well in line with our previous data [27]; here the protection is probably mainly antibody-mediated, although we cannot exclude the presence of unknown CD8^+^ T cell epitopes. We demonstrated very strong protection from FV challenge infection and FV-induced disease in immunized mice when the vaccines were administered sequentially; interestingly, the immune responses significantly differed from those induced by the live-attenuated vaccine virus F-MuLV-N in strength and quality, with no detectable GagL_85–93_-specific CD8^+^ T cell response to F-MuLV-N but a superior antibody response compared to the adenovirus-based vaccine (summarized in Fig. 7). Our finding that F-MuLV-N induced only marginal T cell responses suggests that the potent neutralizing antibody response is the main factor that confers protection, and while it has been demonstrated before in transfer experiments that complex immune responses are required for complete protection [10], and that depletion of T cells from immunized mice would abrogate rapid protection from FV challenge infection [86], it could also be demonstrated that the presence of neutralizing antibodies at the time of challenge infection is crucial for sterile protection [33]. The adenovirus-based vaccine on the other hand induced very strong GagL_85–93_-specific CD8^+^ T cells in immunized mice, whereas the antibody response before challenge infection was fairly low. We saw a rapid increase in neutralizing antibodies after FV challenge infection which was probably assisted by the vaccine-induced CD4^+^ T cells, but it has to be assumed that the lack of potent neutralization prior to FV challenge infection prevents complete protection. An advantage of attenuated virus-based vaccines is the high identity with the pathogen against which they are supposed to protect, which holds true also here, as the N-tropic and B-tropic F-MuLV differ only in a few amino acids in the sequence of the capsid protein [87–89]. It is known that retroviral envelope proteins are extensively and intricately glycosylated [90], which is challenging for vaccine design, as incorrect glycosylation patterns, which are likely to occur in a gene-based vaccine but not in the attenuated virus which is replicating at low level in vivo, can lead to the induction of irrelevant antibodies or to obscuring of immunologically relevant sites [91–93].

Numerous different strategies have been employed in the FV model to date, including attenuated F-MuLV or FV [10, 16, 32, 94], replication-defective [34] or inactivated virus [35], protein- and peptide-based vaccines [36–39, 42], cell-based vaccines, and gene-based vaccines such as plasmid DNA-based [43], vaccinia virus-based [44, 46] or adenovirus-based vaccines [26–31]. While comparisons of all these vaccine approaches are complicated by the use of different mouse strains and widely differing FV challenge doses, it can be determined from these studies that the vaccines that conferred strong protection from FV challenge are the attenuated viruses [10, 16, 32, 94], inactivated virus delivered with complete Freund’s adjuvant [35], a peptide-based vaccine delivering F-MuLV-derived peptides in calcium phosphate nanoparticles together with CpG-containing oligodeoxynucleotides as adjuvant [42], and viral vector based vaccines such as an envelope-encoding vaccinia virus [46] or some of the adenovirus-based vectors developed by us [27, 30, 31]. Interestingly, some of these experiments demonstrate that strong protection from FV challenge is indeed possible without significant induction of neutralizing antibodies, which were reported to be undetectable [46] or at very low level before the FV challenge infection [27, 30] or not induced by the vaccine by design [31, 42, 44], and also demonstrated an important role of non-neutralizing binding antibodies [33]. The only vaccine for which complete protection with absence of any virus-producing cells was demonstrated [16], and for which we found robust neutralizing antibody responses after immunization in the experiments shown here, is the attenuated F-MuLV-N. The protection observed in our mouse model in the highly susceptible CB6F1 mice was not actually complete as described for other susceptible hybrid mouse strains, but this is probably attributable to the very high susceptibility to FV-induced disease of the CB6F1 mice and the high challenge dose employed in our experiments, and emphasizes the stringency of our challenge model.

## Conclusions

We demonstrate here an adenovirus-based vaccination approach that mediates robust protection from retrovirus infection in a highly stringent mouse infection model and is comparable in its efficacy to a live-attenuated retrovirus vaccine, although the mechanisms of protection clearly differ. Our results highlight that the order of vaccine delivery is important for the immunization outcome as a retroviral surface envelope protein can exert suppressive effects on simultaneously or subsequently administered immunogens, and pre-existing immunity in Ad-experienced recipients can actually lead to improved transgene-specific antibody responses.

### Additional files

**Additional file 1.** Suppression of OT-I specific CD8^+^ T cells by F-MuLV Env. CB6F1 mice were immunized once with 10^9^ vp Ad.ova, or with Ad.ova in combination with Ad.env. To ensure that mice of both groups were inoculated with an equal amount of viral particles, the Ad.ova group received an additional 10^9^ vp of an empty Ad vector. The ovalbumin-specific CD8^+^ T cell response was analyzed by intracellular cytokine staining after in vitro restimulation of peripheral blood cells two weeks after the immunization. The production of IFNγ by ova_257–264_-peptide restimulated CD8^+^ T cells was significantly lower in mice co-immunized with Ad.env. The data shown were obtained in two independent experiments, each dot represents an individual mouse. Black lines indicate the mean values, the grey line indicates the mean value after outlier exclusion. Data were analyzed for statistically significant difference, # indicates *P* < 0.05. Statistical significance was calculated with and without inclusion of the outlier and was confirmed for both cases (black symbol: inclusion of outlier, Mann–Whitney Rank Sum Test, *P* = 0.002; grey symbol: exclusion of outlier, t-test, *P* = 0.002).

**Additional file 2.** Experimental layout. CB6F1 mice were immunized with 10^9^ vp of Ad.pIXgp70 or Ad.TxnGagL, or Ad.GFP as a control, in week 0, followed by a second immunization in week 3 with 10^9^ vp of Ad.pIXgp70 or Ad.TxnGagL as indicated. Antibody responses and CD4^+^ and CD8^+^ T cell responses were analyzed at the indicated time points. In week 6, mice were challenged with 5000 SFFU FV, and plasma and spleen viral loads were analyzed at the indicated time points.

**Additional file 3.** Cytokine production by GagL_85–93_-specific CD8^+^ T cells after sequential immunization. CB6F1 mice were immunized at two time points with 10^9^ vp of the indicated vectors; immunizations were performed in a three-week interval, only one vector was used in each immunization. The GagL_85–93_-specific CD8^+^ T cell response was analyzed two weeks after the second immunization by intracellular cytokine staining after restimulation with GagL_85–93_ peptide. The pie charts indicate the mean frequency of CD44^+^ CD8^+^ T cells producing multiple or single cytokines, the arcs around the pies indicate production of the cytokines IFNγ, TNFα and IL2. The size of the pie charts indicates the total frequency of cytokine producing CD44^+^ CD8^+^ T cells, which is also stated below the group names (% cytokine-producing CD44^+^ of CD8^+^ cells). Mean values were calculated from data of 3 mice per group; data were analyzed for statistically significant differences by One Way ANOVA on Ranks and Student–Newman–Keuls post testing, * indicates *P* < 0.05 compared to unvaccinated mice.

**Additional file 4.** Experimental layout. (A) CB6F1 mice were immunized with 10^9^ vp of Ad.pIXgp70 or Ad.Env in week 3, with or without a pre-immunization with an empty Ad vector in week 0. Antibody responses were analyzed in week 5. (B) CB6F1 mice were immunized twice with 10^9^ vp of Ad.empty, one group of mice was immunized with 10^9^ vp of Ad.env in week 6 (upper panel), another group of mice was sacrificed at this time point to collect plasma and CD4^+^ T cells that were transferred either seperately or as an admixture into naïve recipient CB6F1 mice, which were immunized one day later with 10^9^ vp of Ad.Env (lower panel). Antibody responses were analyzed in week 8 (week 2* for transfer recipient mice).

**Additional file 5.** Enhanced binding antibody titers after Ad.leader-gag immunization of Ad pre-immune mice. CB6F1 mice were pre-immunized twice in a three-week interval with 10^9^ vp Ad.empty, and immunized three weeks later with 10^9^ vp Ad.leader-gag, a control group received only the injection of the vaccine vector Ad.leader-gag without pre-immunization. The F-MuLV-binding antibody titers were analyzed in blood samples collected two weeks after the Ad.leader-gag immunization by ELISA. Mice that had been pre-immunized showed significantly higher binding antibody levels than pre-naive mice. Each dot represents an individual mouse, the lines indicate the median values. Data were analyzed for a statistically significant difference by Mann–Whitney Rank Sum Test, # indicates *P* < 0.05. Sufficient statistical power was verified (SP = 0.95).

**Additional file 6.** Repeated Ad pre-immunization leads to unresponsiveness to Ad.TxnGagL immunization. CB6F1 mice were pre-immunized twice in a three-week interval with 10^9^ vp Ad.empty, and immunized three weeks later with 10^9^ vp Ad.TxnGagL, a control group received only the injection of the vaccine vector Ad.TxnGagL without pre-immunization. The Ad5-specific CD8^+^ T cell response (A) and Ad5-binding antibody levels (B) after Ad5 pre-immunization were analyzed two weeks after the second Ad.empty immunization, the GagL_85–93_-specific CD8^+^ T cell response was analyzed two weeks after the Ad.TxnGagL immunization in blood cells by MHC I tetramer staining (C). Mice that had been pre-immunized showed a severe and significant reduction in the frequency of tetramer-binding, GagL_85–93_-specific CD8^+^ T cells. Each dot represents an individual mouse, the lines indicate the mean (A, C) or median values (B), the dotted line indicates the detection limit. Data were analyzed for statistically significant differences by Mann–Whitney Rank Sum Test (A, B), or One Way ANOVA on Ranks and Student–Newman–Keuls post testing (C), * and # indicates *P* < 0.05 compared to unvaccinated mice or pre-naive mice, respectively. Sufficient statistical power was verified (SP = 0.99 (C)).

## References

[1] Pitisuttithum P, Gilbert P, Gurwith M, Heyward W, Martin M, van Griensven F (2006). Randomized, double-blind, placebo-controlled efficacy trial of a bivalent recombinant glycoprotein 120 HIV-1 vaccine among injection drug users in Bangkok, Thailand. J Infect Dis.

[2] Buchbinder SP, Mehrotra DV, Duerr A, Fitzgerald DW, Mogg R, Li D (2008). Efficacy assessment of a cell-mediated immunity HIV-1 vaccine (the Step Study): a double-blind, randomised, placebo-controlled, test-of-concept trial. Lancet.

[3] McElrath MJ, De Rosa SC, Moodie Z, Dubey S, Kierstead L, Janes H (2008). HIV-1 vaccine-induced immunity in the test-of-concept step study: a case-cohort analysis. Lancet.

[4] Rerks-Ngarm S, Pitisuttithum P, Nitayaphan S, Kaewkungwal J, Chiu J, Paris R (2009). Vaccination with ALVAC and AIDSVAX to prevent HIV-1 infection in Thailand. N Engl J Med.

[5] Lavender KJ, Pang WW, Messer RJ, Duley AK, Race B, Phillips K (2013). BLT-humanized C57BL/6 Rag2-/-γc-/-CD47-/- mice are resistant to GVHD and develop B- and T-cell immunity to HIV infection. Blood.

[6] Greenwood EJD, Schmidt F, Kondova I, Niphuis H, Hodara VL, Clissold L (2015). Simian Immunodeficiency virus infection of chimpanzees (Pan troglodytes) shares features of both pathogenic and non-pathogenic lentiviral infections. PLoS Pathog.

[7] Akkina R (2013). Human immune responses and potential for vaccine assessment in humanized mice. Curr Opin Immunol.

[8] Wyand MS, Manson KH, Garcia-Moll M, Montefiori D, Desrosiers RC (1996). Vaccine protection by a triple deletion mutant of simian immunodeficiency virus. J Virol.

[9] Wyand MS, Manson K, Montefiori DC, Lifson JD, Johnson RP, Desrosiers RC (1999). Protection by live, attenuated simian immunodeficiency virus against heterologous challenge. J Virol.

[10] Dittmer U, Brooks DM, Hasenkrug KJ (1999). Requirement for multiple lymphocyte subsets in protection by a live attenuated vaccine against retroviral infection. Nat Med.

[11] Adnan S, Reeves RK, Gillis J, Wong FE, Yu Y, Camp JV (2016). Persistent low-level replication of SIVΔnef drives maturation of antibody and CD8 T cell responses to induce protective immunity against vaginal SIV infection. PLoS Pathog..

[12] Dittmer U, Stahl-Hennig C, Hunsmann G (1997). Live HIV vaccines–how safe?. Nat Med.

[13] Dittmer U, Nisslein T, Meyerhans A, Hunsmann G, Stahl-Hennig C (1997). No reactivation of attenuated immunodeficiency viruses in rhesus macaques after vaccinia virus-induced immune activation. J Gen Virol.

[14] Wyand MS, Manson KH, Lackner AA, Desrosiers RC (1997). Resistance of neonatal monkeys to live attenuated vaccine strains of simian immunodeficiency virus. Nat Med.

[15] Fauci AS, Fischinger PJ (1988). The development of an AIDS vaccine: progress and promise. Public Health Rep.

[16] Dittmer U, Brooks DM, Hasenkrug KJ (1999). Protection against establishment of retroviral persistence by vaccination with a live attenuated virus. J Virol.

[17] Coffin JM (1995). HIV population dynamics in vivo: implications for genetic variation, pathogenesis, and therapy. Science.

[18] Streeck H (2015). AIDS virus seeks refuge in B cell follicles. Nat Med.

[19] Fukazawa Y, Lum R, Okoye AA, Park H, Matsuda K, Bae JY (2015). B cell follicle sanctuary permits persistent productive simian immunodeficiency virus infection in elite controllers. Nat Med.

[20] Whitney JB, Hill AL, Sanisetty S, Penaloza-MacMaster P, Liu J, Shetty M (2014). Rapid seeding of the viral reservoir prior to SIV viraemia in rhesus monkeys. Nature.

[21] Hansen SG, Ford JC, Lewis MS, Ventura AB, Hughes CM, Coyne-Johnson L (2011). Profound early control of highly pathogenic SIV by an effector memory T-cell vaccine. Nature.

[22] Hansen SG, Piatak M, Ventura AB, Hughes CM, Gilbride RM, Ford JC (2013). Immune clearance of highly pathogenic SIV infection. Nature.

[23] Hansen SG, Wu HL, Burwitz BJ, Hughes CM, Hammond KB, Ventura AB (2016). Broadly targeted CD8^+^ T cell responses restricted by major histocompatibility complex E. Science.

[24] Hansen SG, Sacha JB, Hughes CM, Ford JC, Burwitz BJ, Scholz I (2016). Cytomegalovirus vectors violate CD8^+^ T cell epitope recognition paradigms. Science.

[25] Friend C (1957). Cell-free transmission in adult Swiss mice of a disease having the character of a leukemia. J Exp Med.

[26] Bayer W, Schimmer S, Hoffmann D, Dittmer U, Wildner O (2008). Evaluation of the friend virus model for the development of improved adenovirus-vectored anti-retroviral vaccination strategies. Vaccine.

[27] Bayer W, Tenbusch M, Lietz R, Johrden L, Schimmer S, Uberla K (2010). Vaccination with an adenoviral vector that encodes and displays a retroviral antigen induces improved neutralizing antibody and CD4^+^ T-cell responses and confers enhanced protection. J Virol.

[28] Bayer W, Lietz R, Ontikatze T, Johrden L, Tenbusch M, Nabi G (2011). Improved vaccine protection against retrovirus infection after co-administration of adenoviral vectors encoding viral antigens and type I interferon subtypes. Retrovirology.

[29] Lietz R, Bayer W, Ontikatze T, Johrden L, Tenbusch M, Genannt Storcksdieck, Bonsmann M (2012). Codelivery of the chemokine CCL3 by an adenovirus-based vaccine improves protection from retrovirus infection. J Virol.

[30] Ohs I, Windmann S, Wildner O, Dittmer U, Bayer W (2013). Interleukin-encoding adenoviral vectors as genetic adjuvant for vaccination against retroviral infection. PLoS ONE.

[31] Godel P, Windmann S, Dietze KK, Dittmer U, Bayer W (2012). Modification of one epitope-flanking amino acid allows for the induction of friend retrovirus-specific CD8^+^ T cells by adenovirus-based immunization. J Virol.

[32] Dittmer U, Brooks DM, Hasenkrug KJ (1998). Characterization of a live-attenuated retroviral vaccine demonstrates protection via immune mechanisms. J Virol.

[33] Messer RJ, Dittmer U, Peterson KE, Hasenkrug KJ (2004). Essential role for virus-neutralizing antibodies in sterilizing immunity against Friend retrovirus infection. Proc Natl Acad Sci USA.

[34] Ruan KS, Lilly F (1992). Approach to a retrovirus vaccine: immunization of mice against Friend virus disease with a replication-defective Friend murine leukemia virus. Proc Natl Acad Sci USA.

[35] Ishihara C, Miyazawa M, Nishio J, Azuma I, Chesebro B (1992). Use of low toxicity adjuvants and killed virus to induce protective immunity against the Friend murine leukaemia retrovirus-induced disease. Vaccine.

[36] Kleiser C, Schneider J, Bayer H, Hunsmann G (1986). Immunoprevention of Friend leukaemia virus-induced erythroleukaemia by vaccination with aggregated gp70. J Gen Virol.

[37] Ishihara C, Miyazawa M, Nishio J, Chesebro B (1991). Induction of protective immunity to Friend murine leukemia virus in genetic nonresponders to virus envelope protein. J Immunol.

[38] Iwanami N, Niwa A, Yasutomi Y, Tabata N, Miyazawa M (2001). Role of natural killer cells in resistance against friend retrovirus-induced leukemia. J Virol.

[39] Kawabata H, Niwa A, Tsuji-Kawahara S, Uenishi H, Iwanami N, Matsukuma H (2006). Peptide-induced immune protection of CD8^+^ T cell-deficient mice against Friend retrovirus-induced disease. Int Immunol.

[40] Miyazawa M, Fujisawa R, Ishihara C, Takei YA, Shimizu T, Uenishi H (1995). Immunization with a single T helper cell epitope abrogates Friend virus-induced early erythroid proliferation and prevents late leukemia development. J Immunol.

[41] Reuter T, Heldmann M, Schimmer S, Schepers K, Dittmer U (2004). Protection of mice against Friend retrovirus infection by vaccination with antigen-loaded, spleen-derived dendritic cells. Vaccine.

[42] Knuschke T, Bayer W, Rotan O, Sokolova V, Wadwa M, Kirschning CJ (2014). Prophylactic and therapeutic vaccination with a nanoparticle-based peptide vaccine induces efficient protective immunity during acute and chronic retroviral infection. Nanomedicine.

[43] Dittmer U, Werner T, Kraft AR (2008). Co-immunization of mice with a retroviral DNA vaccine and GITRL-encoding plasmid augments vaccine-induced protection against retrovirus infection. Viral Immunol.

[44] Miyazawa M, Nishio J, Chesebro B (1992). Protection against Friend retrovirus-induced leukemia by recombinant vaccinia viruses expressing the gag gene. J Virol.

[45] Hasenkrug KJ, Brooks DM, Nishio J, Chesebro B (1996). Differing T-cell requirements for recombinant retrovirus vaccines. J Virol.

[46] Earl PL, Moss B, Morrison RP, Wehrly K, Nishio J, Chesebro B (1986). T-lymphocyte priming and protection against Friend leukemia by vaccinia-retrovirus env gene recombinant. Science.

[47] Lander MR, Chattopadhyay SK (1984). A Mus dunni cell line that lacks sequences closely related to endogenous murine leukemia viruses and can be infected by ectropic, amphotropic, xenotropic, and mink cell focus-forming viruses. J Virol.

[48] Robertson MN, Miyazawa M, Mori S, Caughey B, Evans LH, Hayes SF (1991). Production of monoclonal antibodies reactive with a denatured form of the Friend murine leukemia virus gp70 envelope protein: use in a focal infectivity assay, immunohistochemical studies, electron microscopy and western blotting. J Virol Methods.

[49] Varghese R, Mikyas Y, Stewart PL, Ralston R (2004). Postentry neutralization of adenovirus type 5 by an antihexon antibody. J Virol.

[50] Perryman S, Nishio J, Chesebro B (1991). Complete nucleotide sequence of Friend murine leukemia virus, strain FB29. Nucleic Acids Res.

[51] Morris JC, Wildner O (2000). Therapy of head and neck squamous cell carcinoma with an oncolytic adenovirus expressing HSV-tk. Mol Ther.

[52] Tenbusch M, Kuate S, Tippler B, Gerlach N, Schimmer S, Dittmer U (2008). Coexpression of GM-CSF and antigen in DNA prime-adenoviral vector boost immunization enhances polyfunctional CD8^+^ T cell responses, whereas expression of GM-CSF antigen fusion protein induces autoimmunity. BMC Immunol.

[53] He TC, Zhou S, da Costa LT, Yu J, Kinzler KW, Vogelstein B (1998). A simplified system for generating recombinant adenoviruses. Proc Natl Acad Sci USA.

[54] Mittereder N, March KL, Trapnell BC (1996). Evaluation of the concentration and bioactivity of adenovirus vectors for gene therapy. J Virol.

[55] Chesebro B, Wehrly K, Stimpfling J (1974). Host genetic control of recovery from Friend leukemia virus-induced splenomegaly: mapping of a gene within the major histocompatability complex. J Exp Med.

[56] Sitbon M, Nishio J, Wehrly K, Lodmell D, Chesebro B (1985). Use of a focal immunofluorescence assay on live cells for quantitation of retroviruses: distinction of host range classes in virus mixtures and biological cloning of dual-tropic murine leukemia viruses. Virology.

[57] Iwashiro M, Kondo T, Shimizu T, Yamagishi H, Takahashi K, Matsubayashi Y (1993). Multiplicity of virus-encoded helper T-cell epitopes expressed on FBL-3 tumor cells. J Virol.

[58] Chen W, Qin H, Chesebro B, Cheever MA (1996). Identification of a gag-encoded cytotoxic T-lymphocyte epitope from FBL-3 leukemia shared by Friend, Moloney, and Rauscher murine leukemia virus-induced tumors. J Virol.

[59] McKelvey T, Tang A, Bett AJ, Casimiro DR, Chastain M (2004). T-cell response to adenovirus hexon and DNA-binding protein in mice. Gene Ther.

[60] Messer RJ, Lavender KJ, Hasenkrug KJ (2014). Mice of the resistant H-2(b) haplotype mount broad CD4(+) T cell responses against 9 distinct Friend virus epitopes. Virology.

[61] Faul F, Erdfelder E, Buchner A, Lang AG (2009). Statistical power analyses using G*Power 3.1: tests for correlation and regression analyses. Behav Res Methods.

[62] Ahi YS, Bangari DS, Mittal SK (2011). Adenoviral vector immunity: its implications and circumvention strategies. Curr Gene Ther.

[63] Varghese R, Mikyas Y, Stewart PL, Ralston R (2004). Postentry neutralization of adenovirus type 5 by an antihexon antibody. J Virol.

[64] Denner J (2014). The transmembrane proteins contribute to immunodeficiencies induced by HIV-1 and other retroviruses. AIDS.

[65] Denner J, Eschricht M, Lauck M, Semaan M, Schlaermann P, Ryu H (2012). Modulation of cytokine release and gene expression by the immunosuppressive domain of gp41 of HIV-1. PLoS ONE.

[66] Toapanta FR, Craigo JK, Montelaro RC, Ross TM (2007). Reduction of anti-HIV-1 Gag immune responses during co-immunization: immune interference by the HIV-1 envelope. Curr HIV Res.

[67] Valentin A, Li J, Rosati M, Kulkarni V, Patel V, Jalah R (2015). Dose-dependent inhibition of Gag cellular immunity by Env in SIV/HIV DNA vaccinated macaques. Hum Vaccin Immunother.

[68] Bockl K, Wild J, Bredl S, Kindsmuller K, Kostler J, Wagner R (2012). Altering an artificial Gagpolnef polyprotein and mode of ENV co-administration affects the immunogenicity of a clade C HIV DNA vaccine. PLoS ONE.

[69] Clutton G, Carpov A, Parks CL, Dean HJ, Montefiori DC, Hanke T (2014). Optimizing parallel induction of HIV type 1-specific antibody and T-cell responses by multicomponent subunit vaccines. AIDS.

[70] Hartman ZC, Kiang A, Everett RS, Serra D, Yang XY, Clay TM (2007). Adenovirus infection triggers a rapid, MyD88-regulated transcriptome response critical to acute-phase and adaptive immune responses in vivo. J Virol.

[71] Hemmi M, Tachibana M, Tsuzuki S, Shoji M, Sakurai F, Kawabata K (2014). The early activation of CD8^+^ T cells is dependent on type I IFN signaling following intramuscular vaccination of adenovirus vector. BioMed Res Int.

[72] Molinier-Frenkel V, Lengagne R, Gaden F, Hong SS, Choppin J, Gahery-Segard H (2002). Adenovirus hexon protein is a potent adjuvant for activation of a cellular immune response. J Virol.

[73] Nociari M, Ocheretina O, Schoggins JW, Falck-Pederson E (2007). Sensing infection by adenovirus: toll-like receptor-independent viral DNA recognition signals activation of the interferon regulatory factor 3 master regulator. J Virol.

[74] Kron MW, Engler T, Schmidt E, Schirmbeck R, Kochanek S, Kreppel F (2011). High-capacity adenoviral vectors circumvent the limitations of dE1 and dE1/dE3 adenovirus vectors to induce multispecific transgene product-directed CD8 T-cell responses. J Gene Med.

[75] Schirmbeck R, Reimann J, Kochanek S, Kreppel F (2008). The immunogenicity of adenovirus vectors limits the multispecificity of CD8 T-cell responses to vector-encoded transgenic antigens. Mol Ther.

[76] Provine NM, Larocca RA, Aid M, Penaloza-MacMaster P, Badamchi-Zadeh A, Borducchi EN (2016). Immediate dysfunction of vaccine-elicited CD8^+^ T cells primed in the absence of CD4^+^ T cells. J Immunol.

[77] Provine NM, Badamchi-Zadeh A, Bricault CA, Penaloza-MacMaster P, Larocca RA, Borducchi EN (2016). Transient CD4^+^ T cell depletion results in delayed development of functional vaccine-elicited antibody responses. J Virol.

[78] Provine NM, Larocca RA, Penaloza-MacMaster P, Borducchi EN, McNally A, Parenteau LR (2014). Longitudinal requirement for CD4^+^ T cell help for adenovirus vector-elicited CD8^+^ T cell responses. J Immunol.

[79] Daly LM, Johnson PA, Donnelly G, Nicolson C, Robertson J, Mills KH (2005). Innate IL-10 promotes the induction of Th2 responses with plasmid DNA expressing HIV gp120. Vaccine.

[80] Tatsis N, Ertl HC (2004). Adenoviruses as vaccine vectors. Mol Ther.

[81] de Andrade Pereira B, Bouillet LEM, Dorigo NA, Fraefel C, Bruna-Romero O (2015). Adenovirus specific pre-immunity induced by natural route of infection does not impair transduction by adenoviral vaccine vectors in mice. PLoS ONE.

[82] Sumida SM, Truitt DM, Kishko MG, Arthur JC, Jackson SS, Gorgone DA (2004). Neutralizing antibodies and CD8^+^ T lymphocytes both contribute to immunity to adenovirus serotype 5 vaccine vectors. J Virol.

[83] Xiang Z, Gao G, Reyes-Sandoval A, Cohen CJ, Li Y, Bergelson JM (2002). Novel, chimpanzee serotype 68-based adenoviral vaccine carrier for induction of antibodies to a transgene product. J Virol.

[84] Saxena M, Van TT, Baird FJ, Coloe PJ, Smooker PM (2013). Pre-existing immunity against vaccine vectors–friend or foe?. Microbiology.

[85] Saxena M, Coloe PJ, Smooker PM (2009). Influence of promoter, gene copy number, and preexisting immunity on humoral and cellular responses to a vectored antigen delivered by a Salmonella enterica vaccine. Clin Vaccine Immunol.

[86] Dittmer U, Race B, Hasenkrug KJ (1999). Kinetics of the development of protective immunity in mice vaccinated with a live attenuated retrovirus. J Virol.

[87] Kozak CA, Chakraborti A (1996). Single amino acid changes in the murine leukemia virus capsid protein gene define the target of Fv1 resistance. Virology.

[88] Lassaux A, Sitbon M, Battini JL (2005). Residues in the murine leukemia virus capsid that differentially govern resistance to mouse Fv1 and human Ref1 restrictions. J Virol.

[89] Ou CY, Boone LR, Koh CK, Tennant RW, Yang WK (1983). Nucleotide sequences of gag-pol regions that determine the Fv-1 host range property of BALB/c N-tropic and B-tropic murine leukemia viruses. J Virol.

[90] Geyer R, Dabrowski J, Dabrowski U, Linder D, Schluter M, Schott HH (1990). Oligosaccharides at individual glycosylation sites in glycoprotein 71 of Friend murine leukemia virus. Eur J Biochem.

[91] Louder MK, Sambor A, Chertova E, Hunte T, Barrett S, Ojong F (2005). HIV-1 envelope pseudotyped viral vectors and infectious molecular clones expressing the same envelope glycoprotein have a similar neutralization phenotype, but culture in peripheral blood mononuclear cells is associated with decreased neutralization sensitivity. Virology.

[92] Raska M, Takahashi K, Czernekova L, Zachova K, Hall S, Moldoveanu Z (2010). Glycosylation patterns of HIV-1 gp120 depend on the type of expressing cells and affect antibody recognition. J Biol Chem.

[93] Wang W, Nie J, Prochnow C, Truong C, Jia Z, Wang S (2013). A systematic study of the N-glycosylation sites of HIV-1 envelope protein on infectivity and antibody-mediated neutralization. Retrovirology.

[94] Halemano K, Barrett BS, Li SX, Harper MS, Smith DS, Heilman KJ (2013). Fv1 restriction and retrovirus vaccine immunity in Apobec3-deficient 129P2 mice. PLoS ONE.

