# Supplementary figures and images for: Induction of complex immune responses and strong protection against retrovirus challenge by adenovirus-based immunization depends on the order of vaccine delivery
Source: Retrovirology. 2017 Feb 6;14:8. doi: 10.1186/s12977-017-0336-7 (PMC5294899; doi:10.1186/s12977-017-0336-7)

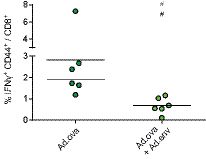

Supplement: Supplementary file 1 — Additional file 1. Suppression of OT-I specific CD8+ T cells by F-MuLV Env. CB6F1 mice were immunized once with 109 vp Ad.ova, or with Ad.ova in combination with Ad.env. To ensure that mice of both groups were inoculated with an equal amount of viral particles, the Ad.ova group received an additional 109 vp of an empty Ad vector. The ovalbumin-specific CD8+ T cell response was analyzed by intracellular cytokine staining after in vitro restimulation of peripheral blood cells two weeks after the immunization. The production of IFNγ by ova257–264-peptide restimulated CD8+ T cells was significantly lower in mice co-immunized with Ad.env. The data shown were obtained in two independent experiments, each dot represents an individual mouse. Black lines indicate the mean values, the grey line indicates the mean value after outlier exclusion. Data were analyzed for statistically significant difference, # indicates P < 0.05. Statistical significance was calculated with and without inclusion of the outlier and was confirmed for both cases (black symbol: inclusion of outlier, Mann–Whitney Rank Sum Test, P = 0.002; grey symbol: exclusion of outlier, t-test, P = 0.002). [file 12977_2017_336_MOESM1_ESM.jpg]

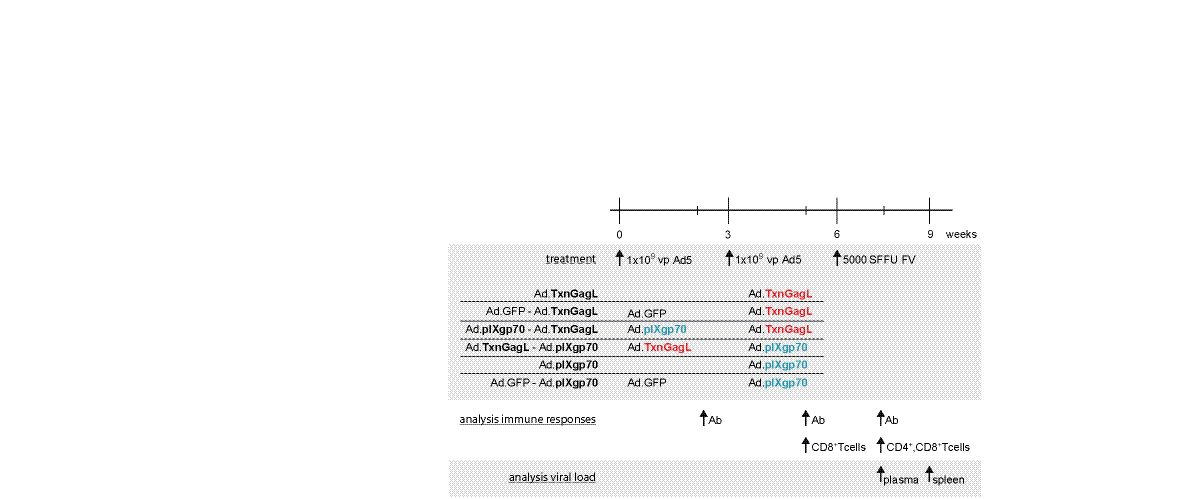

Supplement: Supplementary file 2 — Additional file 2. Experimental layout. CB6F1 mice were immunized with 109 vp of Ad.pIXgp70 or Ad.TxnGagL, or Ad.GFP as a control, in week 0, followed by a second immunization in week 3 with 109 vp of Ad.pIXgp70 or Ad.TxnGagL as indicated. Antibody responses and CD4+ and CD8+ T cell responses were analyzed at the indicated time points. In week 6, mice were challenged with 5000 SFFU FV, and plasma and spleen viral loads were analyzed at the indicated time points. [file 12977_2017_336_MOESM2_ESM.jpg]

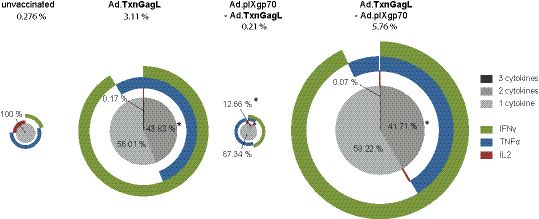

Supplement: Supplementary file 3 — Additional file 3. Cytokine production by GagL85–93-specific CD8+ T cells after sequential immunization. CB6F1 mice were immunized at two time points with 109 vp of the indicated vectors; immunizations were performed in a three-week interval, only one vector was used in each immunization. The GagL85–93-specific CD8+ T cell response was analyzed two weeks after the second immunization by intracellular cytokine staining after restimulation with GagL85–93 peptide. The pie charts indicate the mean frequency of CD44+ CD8+ T cells producing multiple or single cytokines, the arcs around the pies indicate production of the cytokines IFNγ, TNFα and IL2. The size of the pie charts indicates the total frequency of cytokine producing CD44+ CD8+ T cells, which is also stated below the group names (% cytokine-producing CD44+ of CD8+ cells). Mean values were calculated from data of 3 mice per group; data were analyzed for statistically significant differences by One Way ANOVA on Ranks and Student–Newman–Keuls post testing, * indicates P < 0.05 compared to unvaccinated mice. [file 12977_2017_336_MOESM3_ESM.jpg]

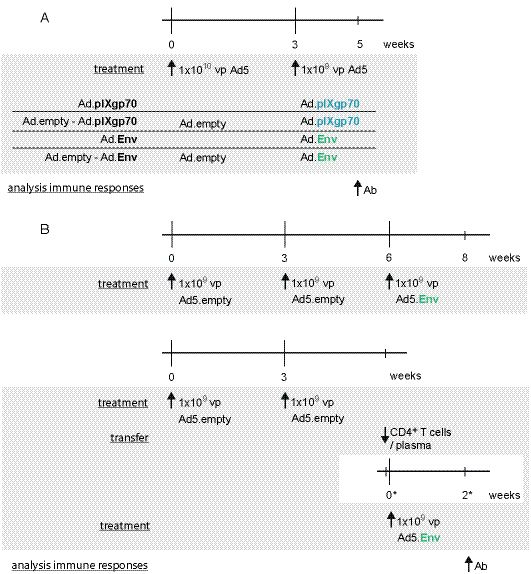

Supplement: Supplementary file 4 — Additional file 4. Experimental layout. (A) CB6F1 mice were immunized with 109 vp of Ad.pIXgp70 or Ad.Env in week 3, with or without a pre-immunization with an empty Ad vector in week 0. Antibody responses were analyzed in week 5. (B) CB6F1 mice were immunized twice with 109 vp of Ad.empty, one group of mice was immunized with 109 vp of Ad.env in week 6 (upper panel), another group of mice was sacrificed at this time point to collect plasma and CD4+ T cells that were transferred either seperately or as an admixture into naïve recipient CB6F1 mice, which were immunized one day later with 109 vp of Ad.Env (lower panel). Antibody responses were analyzed in week 8 (week 2* for transfer recipient mice). [file 12977_2017_336_MOESM4_ESM.jpg]

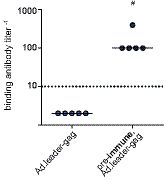

Supplement: Supplementary file 5 — Additional file 5. Enhanced binding antibody titers after Ad.leader-gag immunization of Ad pre-immune mice. CB6F1 mice were pre-immunized twice in a three-week interval with 109 vp Ad.empty, and immunized three weeks later with 109 vp Ad.leader-gag, a control group received only the injection of the vaccine vector Ad.leader-gag without pre-immunization. The F-MuLV-binding antibody titers were analyzed in blood samples collected two weeks after the Ad.leader-gag immunization by ELISA. Mice that had been pre-immunized showed significantly higher binding antibody levels than pre-naive mice. Each dot represents an individual mouse, the lines indicate the median values. Data were analyzed for a statistically significant difference by Mann–Whitney Rank Sum Test, # indicates P < 0.05. Sufficient statistical power was verified (SP = 0.95). [file 12977_2017_336_MOESM5_ESM.jpg]

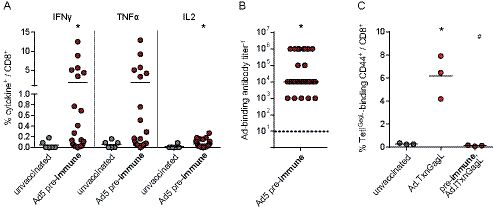

Supplement: Supplementary file 6 — Additional file 6. Repeated Ad pre-immunization leads to unresponsiveness to Ad.TxnGagL immunization. CB6F1 mice were pre-immunized twice in a three-week interval with 109 vp Ad.empty, and immunized three weeks later with 109 vp Ad.TxnGagL, a control group received only the injection of the vaccine vector Ad.TxnGagL without pre-immunization. The Ad5-specific CD8+ T cell response (A) and Ad5-binding antibody levels (B) after Ad5 pre-immunization were analyzed two weeks after the second Ad.empty immunization, the GagL85–93-specific CD8+ T cell response was analyzed two weeks after the Ad.TxnGagL immunization in blood cells by MHC I tetramer staining (C). Mice that had been pre-immunized showed a severe and significant reduction in the frequency of tetramer-binding, GagL85–93-specific CD8+ T cells. Each dot represents an individual mouse, the lines indicate the mean (A, C) or median values (B), the dotted line indicates the detection limit. Data were analyzed for statistically significant differences by Mann–Whitney Rank Sum Test (A, B), or One Way ANOVA on Ranks and Student–Newman–Keuls post testing (C), * and # indicates P < 0.05 compared to unvaccinated mice or pre-naive mice, respectively. Sufficient statistical power was verified (SP = 0.99 (C)). [file 12977_2017_336_MOESM6_ESM.jpg]
